# Collectanea Medica, Consisting of Anecdotes, Facts, Extracts, Illustrations, Queries, Suggestions, &c.

**Published:** 1816-12

**Authors:** 


### COLLECTANEA MEDICA,

CONSISTING OF

### ANECDOTES, FACTS, EXTRACTS, ILLUSTRATIONS, QUERIES, SUGGESTIONS, &c.

RELATING TO THE

*History or the Art of Medicine, and the Auxiliary Sciences.*

Quicquid agunt medici, nostri farrago libelli.

*Observationes Quædam de Hottentotis, præsertim de Structura Genitalium peculiari Hottentotarum.* Auctore GULIELMO SOMERVILLE, M.D. &c. &c.—(From the Medico-Chir. Trans.)

WE do not see the immediate necessity of Latinizing this paper, in a work not likely to circulate among any but medical men. It is, however, desirable that the habit of writing Latin should not be lost among us; and not less so, that we should pay attention to its purity, as often as we use that language. It is no implication to a physician that he is not a classical scholar; but, if he writes Latin, he ought to consult one of that description, and such are always to be found. As we do not pretend to rank ourselves among the number, what follows will, we trust, be considered rather as an enquiry than a critique. After this, we venture to offer two slight objections;—the first, perhaps, scarcely deserves notice. It is to the custom of placing the characteristic mark ^ over the ablatives of the first declensions, when preceded by a preposition governing that case. We are of opinion that a sentence should always be sufficiently perspicuous to render such a mark unnecessary, even without the preposition. This, however, is a mere matter of opinion; but, with a preposition, it is altogether unnecessary, and, as such, improper. Still less are we satisfied with a similar mark over the ablative of the fourth declension, which, as far as our recollection may be depended on, marks the dative, contracted from ui to û.

Another objection we have to make may be called national; yet it is not altogether such, for so many of our young gentlemen are educated in Scotland, or by northern schoolmasters, that Scottisms are becoming more and more frequent in our English, as well as in our Latin, compositions. Most of all, the idiom to which we now wish to direct the reader's attention—we mean the perpetual introduction of the subjunctive mood. In the past tenses, tenses, particularly the imperfect, it has often a classical air. But, without pretending to more than the academic run of scholarship, we will venture to say, that there are few English ears that are not hurt by the continual recurrence of the present of the subjunctive, in the northern writers. What we now offer is chiefly with a wish that some younger graduate, at an age when he is supposed to be disengaged from professional anxieties, would enter minutely on a question which ought to be ascertained, but which, like that of the variety in our English future, most writers seem anxious to avoid. Premising, therefore, that our only object is to gain information, we shall take the first paragraph of this paper for our illustration.

"Omnes fere qui de Promontorio Africæ Australi Capite Bonæ Spei dicto scripserunt, peculiarem partium genitalium Hottentotarum fabricam notârunt: eorum autem scripta adeo inter se discrepant, adeoque obscura sunt, ut non *desint qui dubitent* in qua genitalium parte, insit mira illa varietas; quod oriri potuit vel ex inscitiâ scriptorum, vel ex inutili ipsorum studio, res quas viderant, parum aptâ similitudine illustrandi."

We wish to ask why *desint* and *dubitent* are in the subjunctive mood. We would offer it as a general inquiry, whether the subjunctive mood does not, even in its present tense, always imply something like a contingency almost bordering on futurity. Hence some authors have remarked the close analogy, and almost interchange in the different conjugations, between the future indicative, and the present subjunctive.

The learned author of the Port-Royal Grammar observes, of the conjunction *ut*, that it commonly requires the subjunctive mood, if it has a verb of the present or future tense before it. He afterwards adds, that, even after a preterite verb, the subjunctive imperfect sometimes follows *ut*. In all these cases, we conceive, some contingency is attached to the subjunctive which follows *ut*, as in the examples given by that learned writer; but, in the passage transcribed above, the proper antecedent to *ut* is *adeo*, and the following verbs *desint* and *dubitent*, though placed by the author in the subjunctive mood, are in themselves positive or *indicative*. That such is the meaning of the passage, no one can doubt. For there is no suggestion that from the discrepancy and obscurity of the writings of different authors, there *may* not be wanting *readers;* but it is absolutely and *unconditionally indicated*, from such premises, that the authors themselves, or some other persons, *are* in doubt, &c.

We beg pardon for these apparently unconnected remarks. We trust, however, that, by this time, our readers are satisfied of the necessary connection between accuracy in words and accuracy in reasoning. But our principal wish is, that the subject may be maturely considered in a metropolis enriched by an university, and from which we have a most respectable quarterly production. As the paper which has given rise to this inquiry will come under the notice of our brethren in that part of the world; and, as the

 author, author himself, probably, may have frequent intercourse with the university, or with the metropolis, or with accomplished scholars in both, nothing will be more agreeable to us than an inquiry on both sides, conducted with that impartiality and politeness which has usually distinguished their journal, and which we have endeavoured to preserve in ours.

Having offered these remarks on a subject not strictly medical, we shall reserve a few others for the conclusion of the paper.

"Nobis consilium erit ea tantum modo proferre, quæ ipsi vidimus, de hujus partis generis humani *structurâ*.

"Multum enim Hottentotæ a fœminis vicinarum gentium differunt, vult*û*, colore, vestit*û*, moribus; maxime autem sermone, non solum quôd ad vocabula, verum etiam modo sonos proferendi, qui quantum nos notavimus, nusquam alibi auditur.

"Variarum tribuum hujus gentis in statur*â* maxime notabilis est differentia; ab omnibus vero aliis gentibus adeo differunt, ut ne mutua quidem cum finitimis matrimonia per multa secula, propria vultus lineamenta deleverint.

"Hottentotis plerisque statura aliquanto brevior quam Europæis est.

"Cutis haud multum absimilis est colore folii caduci: sunt etiam quibus totius corporis lividus pallor cadaveris colorem æmuletur, dum apud maxime fuscos genæ nonnihil rubent.

"Caput rotundum parvum: tempora tenuiora, oculi magis distantes quam aliis gentibus observatur: intervallum inter oculos solitâ prominentiâ caret, quam in Europæis dat unguen nasi: oculi canthus interior ellipsin non angulum facit.

"Non alienum a nostro proposito videtur hic notare, Hottentotos ut alios homines rudes visus acumine pollere: nonnulli feras venandi aut hostes effugiendi perpetuâ fere consuetudine, hâc facultate adeo pollebant, ut in campis arenosis vestigia observare possent, ubi aliis nihil omnino appareret: hanc facultatem enim, utpote tum ad victum tum ad salutem ipsam prorsus necessariam assidue exercent, et sic mirum in modum acuunt.

"Nasus valde simus ab origine in fronte usque ad extremam partem: os magnum, saltem longum est, raro tamen hiat ut apud Æthiopes: labra tenuiora quam Æthiopum et nonnihil rosea: dentes candoris eburnei: cutis frontis, prima etiam ætate, rugosa ex conatû assiduo lucem quam maxime excludendi.

"Hottentotorum magis quam Æthiopum capilli texturam lanæ referunt, crescendi autem modo longe absimilis, nam capilli, vel potius pili, vel lana, haud totam capillitii superficiem occupant, sed a locis quibusdam æquis intervallis inter se distantibus prodeunt, eodem fere modo quô setarum fasciculi scopis inseruntur: ubi, quod raro evenit, capilli duorum pollicum longitudinem attingunt, inter se coeunt & implicantur floccorum instar lanæ.

"Aures parvæ, elegantes, interdum etiam ad arbitrium mobiles.

"Huic genti, fasciarum in infantibus, pileorum in ætate provectioribus, nullus usus. Deformitas rarissima est nisi ex casu aliquo.

aliquo. Thorax amplus, corpus erectum, artus torosi et agiliores multo quam facile crediderint quibus vestitus arctior est familiaris.

"Res notatû dignissimæ in Hottentotâ hæ fere sunt: ætate pubere mammæ fiunt longæ, rotundæ ac firmæ, papillæ vix eminent ultra mammæ ipsius circulum, et latiore quam in aliis mulieribus cinguntur. Brevi post pubertatem, magisque dum uterum gerunt, papillæ aliquantum crescunt, nec unquam omnino recedunt: mammæ post partum alterum tertiumve flaccidæ, rugosæ ac pendulæ fiunt, interdum ad inguen usque descendunt, utribus de collo suspensis simillimæ.

"Nates non more solitô leniter rotundæ ad lumbos exsurgunt, sed recte exeunt, quasi corpus antrorsum inclinatum esset. Nec rara exempla Hottentotarum, quibus nates adeo ultra modum prominent, ut vix non credas hanc gentem ita fabricatam ut ore erecto non ambulet. Nates pro reliquo corpore tam magnæ, procul spectanti sane speciem appendicis extraneæ præbent.

"Fabrica vero pudendorum Hottentotarum ab omni alia muliere maxime secernunt. In monte Veneris pubes interdum nulla, plerumque parca et mollis instar lanæ, quæ pro capillis crescit in capite. Os pubis minus teres est minusque obesum quam apud Europæas.

"Ex interiore parte rimæ pudendi, substantia laxa, pendula ac sæpe rugosa descendit, quæ curiosius explorata duplex esse reperitur, ex nymphis productis constans, adeo arcte inter se cohærentibus, ut simplex esse videatur. Nymphæ in quibusdam extra marginem labiorum quinque uncias descendunt.

"Hottentotæ infanti rima adeo hiat ut nymphæ appareant, & puberem circa ætatem paullatim prodeant. Attamen ex maturâ admodum Venere, brevi flaccescunt et tandem marcedæ fiunt et rugosæ. Hottentotis labia exteriora minora sunt et minus prominent quam aliis mulieribus, interdum etiam adeo sunt tenuia ut pene deesse videantur et difficile omnino sit scitû ubi labia desinunt, ubi nymphæ incipiunt.

"Clitoris solito loco sita, angulo nempe ubi nymphæ inter se dividuntur, fabricam peculiarem de quâ agitur, ipsis nymphis inesse monstrat.

"Urethra aliæque partes vicinæ eundem habent situm ac in aliis fœminis: hæ autem omnes ex tenuitate labiorum et montis Veneris, magis prominere videantur.

"Si ullus mos apud Hottentotis valeret quô nymphæ longiores redderentur, vix nobis latuisset. Nil boni autem a nymphis adeo promissis ipsæ Hottentotæ oriri putant, neque iis curæ est aut cordi nymphas longas habere: quibus longissimæ sunt non ideo pulchriores habentur, neque spernuntur quibus sunt maxime curtæ.

"Si quicquid fuisset huic regioni proprium cui rem referre potuissemus, omnium incolarum nymphæ longiores fuissent.

"Quæcunque fuerit origo et causa insolitæ hujus fabricæ naturalium in fœminis Hottentotis, ipsa jamdudum adeo inveteravit, ut neque spes neque ratio ulla supersit, eam penitus indagandi.

"Regionem

"Regionem nescimus unde hæc gens olim migraverit. Verisimile enim est Cafros aliasque a septentrione et oriente vicinas gentes quibus color niger est, Hottentotos a pristinâ ipsorum sede et domicilio pepulisse. Multaque suadent, Cafros aliasque quasdam gentes Africæ, Arabâ satas esse stirpe. Minime autem licet ex paucis quæ hactenus innotuerunt, aliquid statuere de origine et antiquâ variarum gentium historiâ, quæ immensam hanc incolunt continentem, per totam superficiem omnesque suas oras 'leonum aridam nutricem.' Cœlum enim, solum, herbæ, animalia, tum per littora maris Mediterranei, tum ad Bonæ Spei Promontorium, mire inter se similia. Plinius insuper de incolis regionum Africæ a sedibus Hottentotorum longe remotis, hæc verba habet, 'nymphæ aliquando enormes sunt, quare Coptæ et Mauri circumcidunt,'—quibus plane indicat, Coptas Maurosque istis temporibus ab aliis Africæ gentibus ad Septentrionem et ad occidentem incolentibus, discretos fuisse, ipsâ naturalium fabricâ, quâ ut ostendimus, Hottentotæ differunt a fœminis vel maxime vicinarum gentium.

"Ex gente vagâ ab inimicis oppressâ, rebus adversis fractâ, rerum gestarum historia expectanda non est, neque traditionibus eorum siquæ fuerint, fides habenda. Atque pericula & labores iis subeundi, quos scientiæ amor, ad has arenosas regiones visendas, parum adhuc a nobis investigatas, impellere possit, tot ac tanta sunt, ut vix non desperem partes Africæ internas, prius exploratum iri quam adolescentes quidam in Mauritaniâ, hoc consilio instituti, et sermonis rituumque Maurorum periti, rem magnam fuerint aggressi.

"Jam tantum notanda sunt quæ peculiaria cadavere inciso vidimus in Hottentoti corpore: nempe, cranium valde rotundum, malarum ossa altissima, oculi vero orbita Europæorum similis.

"Natium magnitudo ex ingenti adipis massâ inter musculos & integumenta interpositâ oritur, quæ in cadavere quod inspexi, quatuor digitos crassitudine excedebant. Spina Dorsi et pelvis ut in Europæis bene formatis: neque verum os coccygis retrovertitur instar caudæ, ut quidam perhibent.

"Partes genitales internæ fabricam solitam situmque ut in Europæis habent."

The candour and diligence apparent in every part of the above paper, leave us no room to doubt its accuracy. We shall, however, offer a conjecture on the possible cause of certain peculiarities of figure which distinguish particular districts, especially the Hottentots in the southernmost part of Africa, and the Goitres in the northern part of Italy.

First, we may remark, that each is a despised nation, inhabiting a country mountainous in the north, dry and sandy in the south, to which they have probably been driven by the oppression of their enemies, as suggested of the Hottentots by the learned writer of the paper. Is it not then highly probable, that, during the patriarchal condition of the human race, such a variety may have occurred in a single family, and, by seclusion from the rest, when each each required land enough to rear sheep for their support, might have multiplied, and retained the peculiarities of form by constant intermarriages? If this peculiarity was considered a deformity, and such every peculiarity is to which we are unaccustomed, the individuals of each sex who were free from it would undervalue their relations in proportion as they were more or less strongly marked; and hence a gradual seclusion would take place, of such a kind as could scarcely fail to induce a mutual hatred, till the tribe who would become the weakest, from a refusal in all others to communicate with them, must, by degrees, be glad to find refuge in lands of which they were least likely to be dispossessed. It would be no objection to this, if the Hottentots were proud of some peculiarities in their form. It would only prove, what is universally admitted, that beauty and deformity are only relative, and depend very much, like the fashions of ladies, on what we are accustomed to see, or the subjects on whom we see it.

---

*Some Observations concerning the Medical Properties of the Pyrola Umbellata, and the Arbutus Uva Ursi, of Linnæus.* By Professor Smith Barton. Communicated in a Letter to B. C. Brodie, Esq.—(From the same.)

SIR,

In the fifth volume of the Medico-Chirurgical Transactions, there is a paper, by Dr. William Somerville, on the diuretic operation of the Pyrola Umbellata of Linnæus, in which he states that he has not, in any of the books consulted by him, met with any intimations concerning the medical powers of this beautiful little shrub, except the solitary observation of F. Pursh. I beg to refer to my "Collection for an essay towards a Materia Medica of the United States*," for an account of this plant; and I shall now add some of the further observations which I have collected on the same subject, and which I request you to lay before the Society.

There seems to be a good deal of analogy between the effects of the pyrola umbellata, and the uva ursi, as I have mentioned in the account to which I now refer; and in one case of intermittent, under the care of Dr. John S. Mitchell; the urine was increased in quantity, and was of a black colour. But the diuretic effects of this plant have not, I think, in general, been remarked by those who have been much in the habit of employing it in America. Yet it is probable that it is, in part, at least, by virtue of its operation upon the urinary system as a diuretic, that it has been found so highly useful in one of these affections, which I am presently more particularly to mention.

The black colour of the urine of a person who was under the use of the pyrola, is worthy of notice, especially as the late Dr. Heberden

* Third edition. Philadelphia, 1810.

has has recorded nearly a similar appearance in the urine of a patient who was using the uva ursi, a plant, it has already been observed, most closely allied to the American pyrola. The explanation of the fact, in both cases, is rendered the more difficult to me, as my own experiments, and those of several of my pupils, have satisfied me that iron never does manifest itself in the urine of those patients who have been taking even very large doses of the preparations of this metal, and continuing their use for a long time. I know, indeed, that Mr. Lorry has made a contrary assertion. Besides, the patients using the pyrola and the uva ursi had not been taking iron.

It has been intimated by Dr. Somerville, that the pyrola umbellata is an Indian remedy. The plant is, indeed, a principal article in the materia medica of the Indians of North America. But I am not acquainted with all the diseases in which they employ it. Two of these diseases are rheumatism and fever. In both they employ a strong warm decoction of the whole plant, and give it in such large quantities that it does not, and could hardly, fail to excite copious perspiration. By this operation, and by this I presume, principally, the medicine has often been found very useful in the two complaints which I have just mentioned.

Nor has the practice of using the pyrola in fevers been confined to the Indians. The white inhabitants of many parts of North America also employ it. I am assured, on good authority, that it was very extensively employed, and with excellent effect, in many cases of the typhus fever, which, under the appellation of "camp fever," prevailed among the American troops, and carried off great numbers of them, during the time of the revolutionary or first American war. A decoction of the plant was used, and I believe that it was chiefly of service, by exciting perspiration very largely. At present we hear but little of the use of the pyrola in such cases of fever, it having given way to the employment of the eupatorium perfoliatum of Linnæus, which is, perhaps, more entitled to attention. I have made more particular mention of this eupatorium in my Collections, Part First, pages 28, 55; and in Part Second, pages 22, 26.

All my trials and inquiries respecting the pyrola umbellata have convinced me, that it is an important antilithic; not less so than the uva ursi, which, with me, is no mean commendation. For I have, certainly, in my frequent trials of the latter, in cases of nephritis, and especially nephritis calculosa and arthritica, very generally found it eminently useful. It has even seemed (however different may be the explanation of the fact) to favour the expulsion of granules of calculi, an effect which Dr. Hencher, a long time ago, ascribed to various vegetable astringents.

Dr. Somerville, whose paper is chiefly occupied with an account of the diuretic properties of the pyrola umbellata, and its good effects in dropsies, informs us, that the Hurons, and other Indian nations, "use it in gravelly complaints very commonly." This simple intimation is nearly sufficient to convince me, that the whites, or European Americans, derived their first knowledge of the antilithic powers of this plant from the Indians. In truth, notwithstanding all that has been said, by declaimers of a certain description, and by superficial observers, concerning the paucity of the diseases of the American Indians, and of their peculiar exemption from the most grievous maladies, which so often embitter the days of the civilized man, the Indian, or man of America, is by no means exempted from calculous affections. This, indeed, is so far from being the case, that we have already derived from the savages of North and of South America, several of our real or reputed antilithics, such as, not again to mention the pyrola umbellata, the cissampelos pareira, and the xylosteum tataricum. I hope, at no distant period, to communicate to your very respectable Society the free result of my inquiries concerning the diseases and remedies of the Indians of North America.

If future and more extensive trials shall completely confirm the observations of Dr. Somerville, concerning the highly diuretic powers of the pyrola umbellata, there can be no doubt of the propriety of introducing this vegetable into general practice, and of our bestowing upon it a respectable position in the catalogue of remedies for dropsies. Its tonic power, which is mentioned by Dr. Somerville, and for which there is just foundation, may possibly give the American plant some advantages over other diuretics that are in common use, and which possess, in our usual mode of managing them, nothing, or but little, of the robust quality; as for example, digitalis and squill. For it must be obvious to every practitioner in the least conversant with the nature and treatment of different forms of dropsies, that it is often a matter of the first importance to employ, in the intervals between the use of the debilitant diuretics, the *remedia roborantia*, such as some of the purer bitters, quassia, columbo, &c. and even small doses of opium; and what will often, perhaps, be of more importance, the invigorating and happy influence, in such cases, of a sea voyage. The necessity of using even powerful roborants, is especially indicated in the management of those dropsies, whether anasarca, hydrothorax, or ascites, which return periodically, as in the tertiana hydropica, which I have more than once had occasion to see in the United States; and for which the bark, with crystals of tartar, will sometimes be found the best remedy. It is not necessary to pursue this subject to a greater length; though my inclination would lead me to speak of the happy effects of blood-letting, of mild cathartics, and of a vegetable diet, in many cases of dropsy which have fallen under my immediate notice.

Returning more immediately to the pyrola umbellata, I agree with Mr. Pursh, that this plant is not a legitimate species of the genus of pyrola; and, accordingly, a long time ago, I bestowed upon it another generic appellation, associating along with it the pyrola maculata of Linnæus, another American plant, which is sometimes called "rats-bane," in the United States, on the supposition (certainly unfounded) that this plant is a powerful poison.

I have elsewhere mentioned the uses to which the North-American Indians apply the pyrola rotundifolia. See Transactions of the American Philosophical Society, vol. iii.

The pyrola umbellata is one of the plants (not few in number) which are common to the old and new worlds. Whether in the north of Europe, or in the south of Asia, (in both of which it is indigenous,) it is applied to any medical purposes, I know not. Gmelin, in his Flora Siberica, says nothing of its medical qualities.

I am, dear Sir,
With much respect, and with true esteem,
Your friend, &c.

*London; August* 24, 1815. BENJAMIN SMITH BARTON.

To B. C. Brodie, Esq.

---

*Ergot, from the same American Journal, concluded.*

"THE fatal celebrity of the spotted fever, so called in the United States, has excited some curiosity as to its origin. By some, this fever was at first vaguely attributed to comets; and by others, it has for some years past been as vaguely ascribed to ergot, or spurred rye. No one is to be censured for vigilance in suspicions in these cases; but an equal activity is to be desired in the search of proofs.

"The present moment is not the first in which ergot has been charged with producing extensive diseases; since the charge has been carried back to some events which occurred above two centuries ago.

"First, ergot or spurred rye has been accused of producing dry gangrene, as it has been called; one of the most singular maladies which has yet appeared among the human race. The first attacks of this disease are said to be usually seen in the season of harvest, and for a few months afterwards; and the spurred grain which some have considered as its origin, is said to occur most in wet seasons. The dry gangrene, in its very worst forms, begins by destroying the flesh, ligaments, and bones of the limbs, especially in the lower extremities, and it may chance also to destroy the nose; leaving the patient, should he live thus far, with only his trunk and head; which of course do not long survive this loss of their appendages. There is commonly little or no moisture attending this gangrene, and still less bleeding; the decay being somewhat like that of a tree when rotting.—This disease is often mentioned, when spotted fever is ascribed to ergot; but there seems no resemblance between the two effects thus ascribed to one common cause. The spotted fever when it first arises spontaneously as an epidemic, begins its course commonly (in the northern parts of the United States) in January or February; its attacks often last through the spring, extending sometimes even into the summer and fall: its most genuine form being usually best seen in dry air and dry places. We need not add a detail of the symptoms of the spotted fever, to show their disagreement with those of the dry gangrene; especially as enough to decide the question may be learned from this single remark; namely, that dry gangrene produces little fever, and that spotted fever, when well treated, produces little gangrene. It is not therefore from any analogy between the two complaints that the rise of the one is to be considered as establishing the nature of the other.

"Again: Ergot has been supposed to generate a most violent spasmodic disease. Others, it is true, doubt the truth of this imputation; and yet it is remarkable, that this disease has more affinity, than gangrene has, with spotted fever; and therefore in justice it ought to be more fully characterized; in which case it might perhaps be described as a malignant nervous fever, accompanied with spasm; but not commonly (as some have thought) intermittent; and, in the opinion of some, it has been free from contagion. —But allowing even a perfect identity between this disease and spotted fever in essentials, yet unfortunately in our day we are not furnished with the proper means of precisely ascertaining what produced this spasmodic disease in any of the instances recorded from former times; so that it will be more easy to search at the present moment for proofs, both positive and negative, which shall connect together ergot and spotted fever; than to attempt to demonstrate the same more circuitously. Evidence such as is required in the exact sciences is not to be looked for; but where the life of man is concerned, the spirit of the age demands something like experiment and observation, instead of careless guesses.

"One modern experiment with ergot, which has now become of some note, is ready to our hands; but it is not very convincing as to the specific power of ergot to produce spotted fever. Ergot has lately been given medicinally (but with peculiar precautions) to produce speedy delivery in certain cases of female labour; and others, besides women, have made trial of it for other purposes. It is not yet generally known that any other conclusions are to be drawn from what has thus occurred, than the following, viz. that moderate doses of ergot have no marked effect upon the human race, except in the case of women; and that in them, it has acted only upon one organ; and upon that organ suddenly, and only when it is in a peculiar state. These are certainly very limited grounds for a general conclusion; nevertheless, if we take farther into our account the observations and experiments of former times, we ought not to be surprised, if it should be found, that a large and continued use of ergot in the food of man or of other animals, modifies, if it does not create certain diseases; and that a tendency both to gangrene and to spasm may be among the evils which it thus produces. But that it generates spotted fever specifically, is at present matter of almost pure assumption; particularly as we know that many distempers at times affect one portion or other of our vegetable food, without any notable epidemic disease being observed to follow in the human race. If it be at any time the cause of spotted fever, it must often originate thus in a few only, and be propagated to many others who do not eat rye; which is ascribing more of contagion to the disease, than some will admit.

"Such however is the defective state of our knowledge as to the causes of epidemic diseases, that it becomes medical men to institute rigorous inquiries into these subjects. The events of the animal world in its several branches, as well as of the vegetable world, will in this case require to be watched, and compared with what happens within ourselves. At the same time we ought to be prepared with better tests for the examination of the atmosphere which we breathe and which surrounds us, than the present state of our eudiometers affords us; before we can make satisfactory estimates of every thing which influences the human constitution.

"On the whole we shall observe on this subject, 1st, that wheat is very rarely troubled with ergot, and still more rarely barley; the chief connection of ergot being with rye. 2d, A diligent observer (M. Aymer) has conjectured that smut (charbon) is the parallel disease in wheat, to ergot in rye. 3d, We are morally certain, that rye has never been used in a spurred state or even at all, in many instances where spotted fever and even dry gangrene have appeared: so that these cases must either have been the result of contagion, or have proceeded from some other cause. 4th, In a celebrated case of dry gangrene, namely, that of the Downings, of which more will be said in a note, the only grain employed was damaged wheat; and, if this alone occasioned the disease, it produced it, as it were, in a moment, after a long period of inactivity; and this particular disease, in this case as well as in every other, seems to have been free from contagion. 5th, Those who consider ergot from rye as generating epidemic disease, yet admit that in general its noxious qualities, under common circumstances, last only for a few months. 6th, No disease is known to have been produced by ergot, unless where the ergot has been taken to a large amount, or during a given period. 7th, Though it should be denied, contrary to evidence, that ergot is the sole cause of any given disease; yet ergot may at least prepare the way for a given disease, or may be the cause of one or more of its symptoms. For example; spasm may possibly in some instances be a symptom introduced by ergot; since spasm appeared in the spasmodic fever above noticed, and is the precise effect produced when ergot is used medicinally with women in labour.

"*February* 1816.

"P. S.—That spotted fever is not to be held as the necessary produce of ergot in rye, has been stated above; but ergot is now charged with sometimes producing bowel complaints in cities, and not spotted fever. But why do some attribute spotted fever to ergot, in certain country places? Is it because a constitution of things, more potent than ergot generates this disease, or assists in generating it? In France, the occurrence of spotted fever seems not to have been at all frequent, and in proportion to the abundance of ergot with which it has been infested. The Italians have had petechial fever; and yet do not seem to have complained of the the prevalence of ergot, like the French. The Germans and English also have at times had spotted fever; but have not always laid the blame of it on ergot.

"From these and many other circumstances, we may be disposed to believe with Sydenham, that there are epidemic 'constitutions' or tendencies, which come and go (like ergot itself); and depend on great, but sometimes hidden, causes; and which, as they have withdrawn the plague heretofore from England, seem now to be withdrawing typhus from that country; perhaps in order to introduce spotted fever, to which, as we have just observed, it has by no means been entirely a stranger.

"*March* 1816.

"*Note.*—The search into the history of ergot as connected with disease, and especially with epidemic disease, is not to be made without labour.

"The following are some of the authorities which have been consulted on the present occasion.

"Memoirs of the French Academy for 1666, 10, 561, (being for the year 1676);—1710, H.* 61;—1748, 128;—1759, H. 118;—1763, H. 53;—1769, H. 77. Also, *Memoirs des Savans Etrangers* of the same academy, 2, 53; 3, 68; and 4, 371 and 382. Also, London Phil. Trans. for 1762, p. 523, 526, 529, and 584;—and for 1765, p. 108. Also, Dr. Thomas Bateman on Cutaneous Diseases, 3d edition, p. 134, with the notes. Also, the article Ergot in Dr. Rees's new Cyclopedia, (written by Dr. Bateman, as stated in the preceding work, p. 237.) Lastly, a Dissertation on the Natural History and Medicinal effects of the Secale Cornutum or Ergot; by Dr. Oliver Prescott, of Massachusetts.

"The Memoirs of the Royal Society of Medicine at Paris, vol. 1, (containing Memoirs by Messrs. Jussieu, Poulet, Saillant, and the Abbé Tessier,) have not been inspected; nor yet the work of the celebrated economist, Quesnay, on Gangrene.

"It is believed, that if Dr. Bateman will again peruse Tissot's valuable letter to Sir George Baker, in the London Philosophical Transactions for 1765, he will not find that the best accounts of violent spasmodic disease there mentioned, necessarily imply its connection with want of food.

"With respect to the Downing family, a father, mother, and five children, lost eleven out of twelve feet or legs diseased among them; one of the children actually dying. No notice is taken here of the child at the breast, who died after some weeks, (as the nurse said,) with blackened legs; since it does not appear that it partook of the family diet. The following is a part of the statement of Mr. Bonus, the clergyman of the place, made in reply to the queries of Sir George Baker, M.D. 'We have no rye. This family have been used to buy two bushels of clog-wheat, or rivets,

---

"* H. means here the Historical part of the work, which is distinct from the Memoirs.

or bearded wheat, (as it is variously called in Suffolk,) every fortnight: of this they have made their household bread. This wheat they have bought of a farmer with whom I lodge; who tells me that last year he had some wheat laid, which he gathered and thrashed separately, lest it should spoil his samples; not that it was mildewed or grown, but only discoloured and smaller than the other. This damaged wheat he thrashed last Christmas; and then this poor family used no bread but what was made of it; as likewise did the farmer's own family, and some others in the neighbourhood. We observed that it made bad bread, and worse puddings; but I do not find that it disagreed with any body. A labouring man of the parish who had used this bread, was affected with a numbness in both hands, for about four weeks from the 9th of January. His hands were continually cold, and his fingers-ends peeled. One thumb, he says, still remains without any sensation. In this part of the country, there is a great deal of old ewe mutton killed between the 1st of November and January, some of which is very poor and rotten; and is usually sold at three half-pence or perhaps one penny a pound. In December last, this family lived for three weeks at least upon this mutton; of which they bought a quarter at a time (weighing seven or eight pounds) for one shilling.'—p. 530—531. So far Mr. Bonus. Dr. Charles Wollaston, F. R. S., who also was on the spot, writes thus in his second letter. 'I have taken all the pains I could to inquire into the cause of so remarkable a disorder; and Mr. Bonus has made all the inquiry in his power: but we have not been able to find, that there was any thing particular either in their diet or manner of life, to which it could be attributed. The corn, that is, wheat, with which they made their bread, was certainly very bad; it was wheat that had been cut in a rainy season, and had lain on the ground till many of the grains were black and totally decayed: but many other poor families in the same village made use of the same corn, without receiving any injury from it. One man lost the use of his arm for some time, and still imagines himself that he was afflicted with the same disorder as Downing's family; but, by what, I could not learn from him; there seemed to be no reason for this supposition: he is long since perfectly cured.' Thus far Dr. Wollaston. It does not appear that any thing eaten by this family had disagreed with them, except a meal of pickled pork and pease; but this only produced a slight sickness at the stomach in three of the children; and one, if not two, of the family had been taken ill on that day (January 10,) before dinner time. Some little variations will be found in the different accounts; and, in one instance, January 19 is written for January 10, as it stands in two other places: but, generally speaking, the accounts are minute and deserving of credit. Here we must leave the history of this Downing family, which is often referred to by medical men.

"Sir Joseph Banks's account of the diseases of grain by accident, is not at this moment at hand, nor yet that of Fontana; but the following little table is made out by the help of M. Aymer's Memoirs,

Memoirs, in the third and fourth volumes of the Memoirs of the *Savans Etrangers* above cited, and of other data; some of the synonimes only being suppressed.

"Rust:—Rubigo, *Lat.* Stem, leaves, and whole plant attacked.

"Blast:—Ustilago, *Lat.* Fuligine, *Ital.* Nielle or Crulure, *Fr.* Flower attacked.

"Smut:—Caries, *Lat.* Carie, or Charbon, *Fr.* Grain of wheat attacked.

"Spur:—Secale Cornutum, *Lat.* Ergot, Bled corni, *Fr.* Grain of rye attacked.

"It must be observed, that all of these diseases do not appear to have been known to the ancients, or at least not to have been duly described by them in such of their works as have descended to us. It is commonly noticed by writers in modern times, that ergot prevails in other plants besides rye; and, perhaps, its equivalent is sometimes seen to follow the blossoms of certain plums; but this, perhaps, has not been sufficiently ascertained."

We copy the following from the Times Newspaper of November 21; for, though we conceive the report, if not unfounded, at least much exaggerated, yet we feel anxious for some information on the subject in this country, abounding as it does with philosophical farmers.

"A French paper states, that the use of vitiated rye has produced a singular disease, causing cruel ravages in the commune of Baurepaire, department of Isere. The effects of this poison are described as frightful. It acts with great rapidity, even on the strongest men, producing gangrene in all the limbs, which it detaches from the joints in a manner so horrible, that unfortunate creatures have been seen to live in the greatest agonies with only the trunk remaining. In this disease, emetics have been prescribed, followed by antispasmodics, and especially strong doses of opium, the sedative virtues of which have been very useful. The parts threatened are sometimes covered with cloths dipped in decoction of Jesuits bark. Administered internally, this remedy produced no sensible effect. We should suppose, that the rye had been vitiated by the wetness of the harvest."

---

The following remarks on Rabies, though written in a less connected manner than becomes a pathological paper, contains hints which we have thought ought to be recorded.

"*To the Proprietor of the Dublin Free Man's Journal.*

"Sir—Having read some remarks in Saunders, of the 26th inst. upon the frequency of this dreadful visitation, and from the inadequacy of all human skill, as yet manifested, in its cure, I am astonished at the apathy with which mankind survey human affliction—it seems to receive a transitory impression whenever it closely presses on ourselves, but, when the shock is over, we are like our neighbours. I knew a family that had an open well in their gar-

 den, den, their child fell into it and was drowned; but this did not suggest the necessity of railing in the destructive chasm; another child fell into it and was not drowned: and this perhaps was the reason why the well was left open for the reception of the father or mother, or whomsoever God might send that way. About sixteen years since I was a student under Dr. Stokes, in Mercer's Hospital; a case of hydrophobia occurred (and if you will receive it, I shall publish the case). Dr. Stokes, a man whom I would not venture to pourtray for honesty, humanity, and every quality that adorns a man, and suggests the real character of a physician, lest I should daub, in copying, a fine original picture—this gentleman, at the time, had the Clinical Lectureship of Sir Patrick Dunn's Hospital, then held in that house. A case of hydrophobia was brought into it; a girl had been bitten in the foot, and all the symptoms of the malady followed; Dr. Stokes applied the tourniquet upon the thigh, and cut off all nervous communication with the affected part; the girl called for drink, and drank with avidity—the tourniquet was loosed; the horror of liquid returned. The tourniquet was tightened, and the dread of liquid was removed. Upon this Dr. Stokes proposed cutting off the limb. A consultation was called; there were forty-eight hours consumed in discussing the inhumanity of the operation, and from personal hostility to the man, perhaps a great discovery in medicine was lost. Several cases of hydrophobia have occurred lately, the same fooleries that were always used were adopted, and death followed. Dr. Stokes assured me that he was never called into one case of this malady, since the period I mention; although, ask any physician what can he do for the disease, his answer is, I will try certain operations, all of which are perhaps less humane, and equally fatal, as the old cure of smothering between two beds. When I cured puerperal fever by means of turpentine judiciously combined, I did as much as any man who would now cure hydrophobia. I did more, for there is only one case from India recorded of hydrophobia cured, and medicine does not record one case of puerperal fever cured in the stages I always cured it. History does not record a case of trismus nascentum cured, as I never failed to cure it, and as I look upon hydrophobia to be a spasmodic disease, induced by a poison that admits of remedy, (for even one case cured of a million, proves that fact), I have great confidence that the judicious combination of turpentine with proper antispasmodics, and medicines adapted to the fever of the complaint, would alleviate this dreadful malady. I offer myself to assist the physicians who may be next engaged in this disease. If I do not assist them, I am sure I will not make their practice more fatal than it has ever been. I saw Patrick Walsh, who died a few days ago in Mercer's Hospital of this disease, and I flatter myself, had he taken a medicine that I brought him, he would be as certainly relieved as the three patients in Sackville-lane were, that I cured of the equally mortal malady, the nine-day fits. "John Brenan."

"25, *French-street; Sept.* 28, 1816."

CRITICAL

